# Neutropenic Fever in Lung Cancer: Clinical Aspects Related to Mortality and Antibiotic Failure

**DOI:** 10.3779/j.issn.1009-3419.2021.102.36

**Published:** 2021-11-20

**Authors:** Inês RODRIGUES, Luísa NASCIMENTO, Ana Cláudia PIMENTA, Sara RAIMUNDO, Bebiana CONDE, Ana FERNANDES

**Affiliations:** Pulmonology Department, Centro Hospitalar Trás-os-Montes e Alto Douro, Vila Real, Portugal

**Keywords:** Febrile neutropenia, First-line antibiotic failure, Lung neoplasms, Mortality

## Abstract

**Background and objectives:**

Lung cancer (LC) is the leading cause of cancer death.Patients treated with chemotherapy are at risk of developing chemotherapy-induced febrile neutropenia (FN), a potentially life-threatening complication.The aims of this study were (1) to characterize FN admissions of patients with LC in a pulmonology department, and (2) to determine associations between patient profiles, first-line antibiotic failure (FLAF) and mortality.

**Methods:**

Retrospective observational case-series, based on the analysis of medical records of LC patients that required hospitalization due to chemotherapy-induced FN.

**Results:**

A total of 42 cases of FN were revised, corresponding to 36 patients, of which 86.1% were male, with a mean age of 66.71±9.83 years.Most patients had a performance status (PS) equal or less than 1, and metastatic disease was present in 40.5%(*n*=17).Respiratory tract infections accounted for 42.9%(*n*=18) of FN cases, and multidrug-resistant *Staphylococcus aureus* was the most isolated agent.The mortality rate was 16.7%(*n*=7), and the FLAF was 26.2%(*n*=11).Mortality was associated with a PS≥2(*P*=0.011), infection by a Gram-negative agent (*P*=0.001) and severe anemia (*P*=0.048).FLAF was associated with longer hospitalizations (*P*=0.020), PS≥2(*P*=0.049), respiratory infections (*P*=0.024), and infection by a Gram-negative (*P*=0.003) or multidrug-resistant agent (*P*=0.014).

**Conclusions:**

Lower PS, severe anemia, and infections by Gram-negative or multi-resistant agents seem to be associated with worse outcomes in FN patients.

## Introduction

Lung cancer (LC) is the oncologic disease with the highest mortality rate worldwide, and it is responsible for approximately 18% of deaths due to malignant tumors^[1]^. Although LC treatment is undergoing profound modifications, especially in the field of immuno-oncology and targeted therapies^[2]^, combined chemotherapy regimens are still frequently used in the management of the advanced stages of the disease.

Chemotherapy-induced febrile neutropenia (FN) is a potentially lethal complication, and occurs in 10% to 40% of LC patients who undergo chemotherapy^[3, 4]^. Moreover, many of the regimens commonly used in LC treatment are classified as having a high (> 20%) or intermediate (10%-20%) risk of producing FN^[5]^. Nevertheless, prophylactic treatment with antimicrobials is generally not recommended for patients with solid tumors^[6]^.

Risk stratification scales, like The Multinational Association for Supportive Care in Cancer (MASCC) risk index score^[7-9]^ or the Clinical Index of Stable Febrile Neutropenia (CISNE)^[10, 11]^, have been developed to assess the risk of patients with FN to develop serious medical complications, as well as helping with the initial choice of antibiotic therapy^[12]^. While largely used, they show some limitations: (1) they present low sensitivity and positive predictive value to identify high-risk patients^[8, 11]^; (2) they do not account for biochemical markers predictors of infection or organ dysfunction^[13-16]^; (3) they cannot be reliably used to predict bacteremia^[15, 17, 18]^ nor first-line antibiotic failure (FLAF)^[4, 19]^. The latter is of particular importance, as the bacterial aetiology in neutropenic patients with cancer is changing, with an increase in Gram-negative and multidrug resistance organisms; on the other hand, adequate first-line antibiotic regimens are associated with better outcome^[3, 19, 20]^. Thus, it is of paramount importance the attainment of optimal treatment strategies in this setting, especially for an initial approach.

Therefore, the aims of this study were (1) to characterize FN hospitalizations of LC patients in the pneumology ward of a central hospital, and (2) to search for associations between patient profiles, FLAF, and mortality.

## Materials and Methods

We conducted a retrospective case-series study, based on the analysis of the medical records of patients hospitalized at the pneumology ward of a central hospital in Portugal, between January 2009 and June 2017. We revised all the records of patients diagnosed with LC that exhibited chemotherapy-induced FN, which was defined as a single temperature measurement > 38.3 ℃ or a sustained temperature of ≥38 ℃ for over one hour, in the presence of a neutrophil count < 500/mm^3^.

The following data were recorded: gender, age, LC histological type and staging, Eastern Cooperative Oncology Group performance status (PS)^[21]^ at the time of diagnosis, chemotherapy regimen that led to FN, use of radiotherapy, duration and magnitude of FN, hospitalization time and treatment with granulocyte colony-stimulating factors (G-CSF). Concerning the infection, we recorded the site of infection (if known), antibiotic and antifungal therapy used, and microbiological culture and Gram stain. Finally, we also recorded comorbidities, namely a history of chronic respiratory disease, heart disease, or diabetes mellitus, and the presence of the following severity criteria at admission: systolic blood pressure < 90 mmHg, severe thrombocytopenia (platelets < 50×10^3^/mm^3^), severe anemia [hemoglobin (Hb) < 8.0 g/dL] and a creatinine level > 1.2 mg/dL.

A diagnosis of FLAF was made when the outcome did not meet all of the following criteria: (1) no fever for 3 successive days, (2) disappearance of clinical signs of infection, (3) eradication of a known pathogen, (4) no recurrence within 7 days of the end of antibiotic therapy^[22]^. Mortality was defined as any FN-related death within the first 30 days of FN onset.

Categorical variables were expressed as frequency and percentage, and continuous variables as Mean±Standard deviation (SD) or median and interquartile range (IQR) when not normally distributed. Categorical variables were compared using the *Chi-square* or *Fisher's* exact test, and continuous variables were compared using the *Student's t*-test or the *Mann-Whitney U* test. *Haldane-Anscombe* correction was applied when appropriate. Normal distribution was checked using the *Shapiro-Wilk* test or *Skewness and Kurtosis*. The threshold for statistical significance was set to *P* < 0.05. All the statistical procedures were performed using IBM Statistical Package for the Social Sciences (SPSS) software, version 23.0.0.

## Results

We included 42 cases of FN, which occurred in 36 patients (6 patients had 2 episodes of FN). Characteristics of patients and FN episodes are summarized in Table 1. Most of the patients were male (86.1%), and the mean age was 66.71±9.83 years (range 42 to 85). Regarding LC histological type and staging, 27.8% (*n*=10) of the patients were diagnosed with small cell lung cancer (SCLC), and 40.5% (*n*=17) had metastatic disease. About a quarter was treated with a combination of carboplatin/etoposide, 10 (23.8%) with carboplatin/pemetrexed, 6 (14.3%) with carboplatin/vinorelbine, and another 6 with carboplatin/gemcitabine. Most patients presented a good PS, with only 9.5% of the cases (*n*=4) having a PS equal or greater than 2. Active smokers accounted for 26.1% (*n*=11) of all patients. Only 2 cases (4.8%) were under primary G-CSF prophylaxis, and the majority (92.9%, *n*=39) received G-CSF treatment during the FN episode. As for comorbidities, 31.0% (*n*=13) and 26.2% (*n*=11) of the patients had a history of chronic heart and respiratory disease, respectively, and 19.0% (*n*=8) of diabetes mellitus.

**Table 1:** Patient and FN episodes characteristics

Characteristics	*n* (%)/Mean±SD
Gender (Male/Female)	31 (86.1%)/5 (13.9%)
Age (yr)	66.71±9.83
SCLC	10 (27.8%)
Metastatic cancer	17 (40.5%)
PS≥2	4 (9.5%)
Chemotherapy regiments	
Carboplatin/etoposide	11 (26.2%)
Carboplatin/pemetrexed	10 (23.8%)
Carboplatin/vinorelbine	6 (14.3%)
Carboplatin/gemcitabine	6 (14.3%)
Others	9 (21.4%)
Radiotherapy	20 (47.6%)
Active smoker	11 (26.1%)
G-CSF prophylaxis	2 (4.8%)
G-CSF treatment	39 (92.9%)
Comorbidities	
Chronic respiratory disease	11 (26.2%)
Chronic heart disease	13 (31.0%)
Diabetes mellitus	8 (19.0%)
Duration of hospitalization (d)	
Median	9
IQR	7-15.2
Duration of neutropenia (d)	
Median	3
IQR	2-4.5
Neutrophil count (mm^3^)	
Median	185
IQR	100-370
Severity criteria (admission)	
Severe anemia (Hb < 8.0 g/dL)	6 (14.3%)
Severe thrombocytopenia (platelet < 50×10^3^/mm^3^)	10 (23.8%)
Systolic blood pressure < 90 mmHg	11 (26.2%)
Creatinine > 1.2 mg/dL	8 (19.0%)
SCLC: small cell lung cancer; PS: performance status; G-CSF: granulocyte colony-stimulating factors; Hb: hemoglobin.	

The median duration of hospitalization was 9 d, range 3 d to 31 d. The median neutrophil count was 185/mm^3^, and the median duration of neutropenia was 3 d. At admission, 26.9% of the patients (*n*=11) had a systolic blood pressure < 90 mmHg, 23.8% (*n*=10) severe thrombocytopenia, 14.3% (*n*=6) severe anemia and 19.0% (*n*=9) had a creatinine level above 1.2 mg/dL.

Concerning the infection itself (Table 2), respiratory tract infections accounted for 42.9% (*n*=18) of FN cases, digestive tract infections for 16.7% (*n*=7), urinary tract infections for 4.8% (*n*=2) and cellulitis for 2.4% (*n*=1). In 33.3% (*n*=14) of the cases, the infection site remained unknown. Of the 11 patients (26.2%) with identified pathogens, 8 were Gram-positive bacteria (72.7%) and 3 Gram-negative (27.3%). *Methicillin-resistant Staphylococcus aureus* was the only multidrug resistant bacteria isolated, and was also the most isolated organism, corresponding to 7.14% (*n*=3) of all cases. None of the patients were under prophylactic treatment with antibiotic or anti-fungal when they developed FN. Most were initially treated with either a carbapenem (45.2%; *n*=19) or piperacillin-tazobactam (42.9%; *n*=18); 5 patients were treated with a fluoroquinolone (11.9%). An anti-fungal agent was added to the treatment in 15 patients (35.7%). FLAF occurred in 26.2% (*n*=11), and the mortality rate was 16.7% (*n*=7).

**Table 2:** Infection and treatment characteristics

Characteristics	*n* (%)
Infection classification by site	
Respiratory tract	18 (42.9%)
Digestive tract	7 (16.7%)
Urinary tract	2 (4.8%)
Cellulitis	1 (2.4%)
Unknown origin	14 (33.3%)
Microbiological characteristics	
Gram-positive	8 (19.0%)
Gram-negative	3 (7.1%)
No microorganism isolated	31 (73.8%)
Multiresistant organism	3 (7.1%)
Antibiotic used	
Carbapenem	19 (45.2%)
Piperacillin-Tazobactam	18 (42.9%)
Fluoroquinolone	5 (11.9%)
Anti-fungal	15 (35.7%)

Mortality was significantly associated to the following variables (Table 3): PS equal or greater than 2 (*P*=0.011), infection by Gram-negative bacteria (*P*=0.003) and severe anemia (*P*=0.048). FLAF, on the other hand, was associated with younger age (*P*=0.049), longer hospitalizations (*P*=0.021), PS equal or greater than 2 (*P*=0.049), respiratory infection (*P*=0.033), infection by Gram-negative bacteria (*P*=0.014) or multidrug-resistant bacteria (*P*=0.014) and greater use of antifungal (*P*=0.034). Infection by Gram-negative bacteria was associated with both outcomes, with the 3 cases of Gram-negative isolates presenting FLAF (*P*=0.003) and death (*P*=0.014).

**Table 3:** Association between patient characteristics, mortality, and first-line antibiotic failure (FLAF)

	Mortality [*n* (%)]/Mean±SD				FLAF [*n* (%)]/Mean±SD		
	Yes (*n*=7)	No (*n*=35)	*P*		Yes (*n*=11)	No (*n*=31)	*P*
Gender (male)	6 (85.7%)	29 (82.9%)	> 0.999^a^		8 (72.7%)	27 (87.1%)	0.353^a^
Age (yr)	67.00±10.66	66.66±9.83	0.934		61.73±10.42	68.48±1.64	0.049
SCLC	3 (42.9%)	8 (22.9%)	> 0.999^a^		4 (36.4%)	7 (22.6%)	0.437^a^
Metastatic disease	3 (42.9%)	14 (40%)	0.603^a^		3 (27.3%)	14 (45.2%)	0.477^a^
PS≥2	3 (42.9%)	1 (2.9%)	0.011^a^		3 (27.3%)	1 (3.2%)	0.049^a^
Radiotherapy	4 (57.1%)	16 (45.7%)	0.691^a^		5 (45.5%)	15 (48.4%)	0.867
Active smoker	3 (42.9%)	8 (22.9%)	0.353^a^		5 (45.5%)	6 (19.4%)	0.120^a^
Comorbidities							
Chronic respiratory disease	0 (0.0%)	11 (31.4%)	0.161^a^		2 (18.2%)	9 (29%)	0.696^a^
Chronic heart disease	2 (28.6%)	11 (31.4%)	> 0.999^a^		3 (27.3%)	10 (32.3%)	> 0.999^a^
Diabetes mellitus	1 (14.3%)	7 (20.0%)	> 0.999^a^		2 (18.2%)	6 (19.4%)	> 0.999^a^
Severity criteria (admission)							
Severe anemia (Hb < 8.0 g/dL)	3 (42.9%)	3 (8.6%)	0.048^a^		2 (18.2%)	4 (12.9%)	0.644^a^
Severe thrombocytopenia (platelets < 50×10^3^/mm^3^)	3 (42.9%)	7 (20.0%)	0.328^a^		4 (36.4%)	6 (19.4%)	0.410^a^
Systolic heart rate < 90 mmHg	1 (14.3%)	10 (28.6%)	0.654^a^		1 (9.1%)	10 (32.3%)	0.234^a^
Creatinine > 1.2 mg/dL	1 (14.3%)	7 (20.0%)	> 0.999^a^		3 (27.3%)	5 (16.1%)	0.412^a^
Duration of hospitalization (d)							
Median	7	9	0.204		16	8	0.021
IQR	5-13	8-17			11-19	7-10	
Neutrophil count (mm^3^)							
Median	157	190	0.190		180	190	0.179
IQR	100-230	100-400			60-330	120-400	
Duration of neutropenia (d)							
Median	4	3	0.532		4	3	0.616
IQR	3-5	2-4			3-5	2-4	
G-CSF prophylaxis	0 (0.0%)	2 (5.7%)	> 0.999^a^		1 (9.1%)	1 (3.2%)	0.460^a^
G-CSF treatment	7 (100.0%)	32 (91.4%)	> 0.999^a^		10 (91%)	29 (93.5%)	> 0.999^a^
Type of infection and treatment							
Respiratory infection	4 (57.1%)	14 (40.0%)	0.438^a^		8 (72.7%)	10 (32.3%)	0.033^a^
Gram-negative bacteria	3 (42.9%)	0 (0.0%)	0.003^a^		3 (27.3%)	0 (0.0%)	0.014^a^
Positive blood cultures	2 (28.6%)	2 (5.7%)	0.123^a^		2 (18.2%)	2 (6.5%)	0.277^a^
Multidrug resistant bacteria	0 (0.0%)	3 (8.6%)	> 0.999^a^		3 (27.3%)	0 (0.0%)	0.014^a^
Anti-fungal	3 (42.9%)	12 (34.3%)	0.686^a^		7 (63.6%)	8 (25.8%)	0.034^a^
^a^*Fisher's* Exact Test was used.							

## Discussion

As expected, most of our sample consisted of men over 65 years of age. About a quarter had a diagnosis of SCLC and almost half had metastatic disease at the time of the FN episode, but neither of these characteristics appeared related to mortality nor FLAF. The presence of comorbidities also did not have a negative impact on either mortality or FLAF. Similar results can also be found in the studies by Lanoix *et al*^[3]^ and Fujiwara *et al*^[23]^.

Although poor PS is often associated with an increased risk of FN^[4]^, almost 90% of the patients had a PS of 0 or 1. Despite this, PS equal or greater than 2 was associated with both an increase in mortality and FLAF. As verified by Park *et al*^[24]^, pre-admission PS in critically ill patients seems to be an important prognostic factor, regardless of the severity of physiologic derangement, and it's an aspect to consider in assessing risk in FN patients, despite not being evaluated when using the MASCC score.

No statistically significant differences were found between age and mortality, but there was an association between FLAF and younger age (*P*=0.049). A study by Zhai *et al*^[19]^ in patients with haematological malignancies also described higher antibiotic failure rates in patients less than 60 years old, although no statistical significance was reached. Contrary to this, Lanoix *et al*^[3]^ concluded, using a multivariate analysis, that age > 60 years was a risk factor to FLAF in LC patients. These contradictory results may be a consequence of other factors: in our sample, younger patients also had higher PS and a higher rate of infections by multidrug-resistant bacteria, both also associated with FLAF.

Severe anemia was associated with higher mortality, but not with FLAF. Anemia is common in cancer patients, as it may be caused by the disease itself or as a result of chemotherapy's toxicity. It is also associated with shorter survival times in patients with lung carcinoma, according to Caro *et al*^[25]^. Moreover, and despite not being statistically significant in our sample, other studies have also concluded that severe thrombocytopenia^[3, 16]^ and low absolute neutrophil count^[18, 19]^ are risk factors for adverse outcomes in FN, which further highlights the importance of haematological assessment on the prognosis and risk stratification of these patients.

Infection by Gram-negative bacteria was associated with both mortality and FLAF. The subset of patients infected by Gram-negative organisms represents a low proportion of neutropenic patients in our sample (7.1%), which may compromise its significance, but is concordant with the results of several other studies^[5, 18, 19]^. It is also known that Gram-negative bacteria show greater resistance to various antibiotics than Gram-positive bacteria, mainly due to the nature of their cell wall^[26]^. Furthermore, Paesmans *et al*^[18]^ concluded that Gram-negative bacteraemia is significantly more frequent in high-risk patients, and is associated with a very high rate of complications and death as well. Considering this, it seems plausible this group could benefit from risk stratification tools that take Gram stain into account.

We also found statistically significant associations between FLAF and respiratory tract infections, multidrug-resistant bacteria, and antifungal therapy. The first two are probably related, as all multidrug-resistant isolations in our study were obtained from respiratory secretions. Nevertheless, Zhai *et al*^[19]^ and Baskaran *et al*^[27]^ reported worse outcomes in patients with haematological malignancies and FN caused by respiratory infections; this suggests that even in patients with no underlying lung disease, respiratory infections may be associated with FLAF, irrespective of antimicrobial resistance profile.

Regarding antifungal therapy, our results are justified by IDSA guidelines recommendation that "empirical antifungal coverage should be considered in high-risk patients who have a persistent fever after 4 d-7 d of a broad-spectrum antibacterial regimen and no identified fever source"^[12]^, which is compatible with our definition of FLAF.

Finally, mortality and FLAF rates were 16.7% and 26.3%, respectively. Mortality rate was lower than the rate reported by Lanoix *et al*^[3]^ study (33%), but slightly higher than the one obtained by Kuderer *et al*^[28]^ (13.4%), when considering only LC. FLAF rate, on the other hand, was similar to other studies^[3]^.

The fact that our study was conducted in a single centre and had a retrospective design is a manifest limitation to the present work. Also, the small sample size prevented statistical robustness and the application of binary logistic regression tests that could allow further conclusions.

## Conclusions

FN remains an important cause of morbidity and mortality in LC patients. The present work shows that worse PS, severe anemia, and infections by Gram-negative or multi-resistant agents are associated with worse outcomes, even though risk stratification scales do not account for most of these variables. Further studies in this area could possibly lead to the development of better tools for risk stratification, allowing a better management of the condition for high-risk patients, and, hopefully, improved outcomes.
